# FGF-23 serum levels and bone histomorphometric results in adult patients with chronic kidney disease on dialysis 

**DOI:** 10.5414/CN108407

**Published:** 2014-09-10

**Authors:** Florence Lima, Amr El-Husseini, Marie-Claude Monier-Faugere, Valentin David, Hanna Mawad, Darryl Quarles, Hartmut H. Malluche

**Affiliations:** 1 Division of Nephrology, Bone and Mineral Metabolism, University of Kentucky, Lexington, KY,; 2 Division of Nephrology and Hypertension, University of Miami, Miami, FL, and; 3 Division of Nephrology, University of Tennessee, Memphis, TN, USA

**Keywords:** FGF-23, renal osteodystrophy, bone histomorphometry, bone mineralization, dialysis

## Abstract

Background: Fibroblast growth factor-23 (FGF-23) is a hormone principally produced by osteocytes/osteoblasts. In patients with chronic kidney disease (CKD), FGF-23 levels are usually elevated and can reach up to 300 – 400 times the normal range. FGF-23 is regulated by local bone-related and systemic factors, but the relationship between circulating FGF-23 concentrations and bone remodeling and mineralization in CKD has not been well characterized. In the current study, we examined the relationship between FGF-23 levels and bone histomorphometry parameters in adult patients with renal osteodystrophy. Material and methods: 36 patients on dialysis (CKD-5D) underwent bone biopsies after tetracycline double labeling. Blood drawings were done at time of biopsy to determine serum levels of markers of bone and mineral metabolism. Results: Patients with high bone turnover had higher values of serum FGF-23 than patients with low bone turnover. FGF-23 levels correlated with activation frequency (ρ = 0.60, p < 0.01) and bone formation rate (ρ = 0.57, p < 0.01). Normal mineralization was observed in 90% of patients with FGF-23 levels above 2,000 pg/mL. Furthermore, FGF-23 correlated negatively with mineralization lag time (ρ = –0.69, p < 0.01) and osteoid maturation time (ρ = –0.46, p < 0.05) but not with osteoid thickness (ρ = 0.08, ns). Regression analysis showed that FGF-23 was the only independent predictor of mineralization lag time. FGF-23 correlated with cancellous bone volume (ρ = 0.38, p < 0.05) but did not predict it. Conclusion: Circulating FGF-23 concentrations may reflect alterations in ongoing bone formation along with active mineralization, but not exclusively in bone formation or mineralization. Abnormal mineralization lag time (> 100 days) was mainly seen in patients with FGF-23 levels less than 2,000 pg/mL, while very high levels of FGF-23 are associated with normal mineralization lag time.

## Introduction 

Fibroblast growth factor-23 (FGF-23) is produced mainly by osteocytes/osteoblasts in bone [1, 2] and is known to act on the kidney as a “phosphaturic hormone” by inhibiting renal phosphate reabsorption and 1,25-dihydroxyvitamin D (1,25D) production [3, 4, 5]. Moreover, there is emerging evidence linking FGF-23 regulation with bone metabolism. Studies on human genetic and acquired diseases, as well as tissue culture and genetically modified animal models, have demonstrated that both extremely high and low serum FGF-23 levels are associated with skeletal abnormalities due to impaired mineralization [4, 6]. In humans with normal renal function, excess FGF-23 causes rickets/osteomalacia and growth retardation, principally through lower serum phosphorus and suppressing 1,25D levels [7, 8, 9, 10, 11]. Bone mineralization is also impaired in FGF-23 null mouse models, possibly due to the marked excess of 1,25D and elevated osteopontin levels in these mutant mice [12]. Recently, FGF-23 has been purported to have direct effects on osteoblasts in-vitro through activation of FGF receptors/soluble Klotho (s-Klotho) complexes [13]. Also, both local (FGF receptor activation and bone turnover) and systemic factors (i.e., 1,25D, iron, parathyroid hormone (PTH) and calcium) regulate FGF-23 production by osteoblasts [3, 14, 15]. 

In chronic kidney disease (CKD), circulating levels of intact FGF-23 are dramatically increased. This increase starts before the rise in serum intact parathyroid hormone (iPTH) and can reach levels that are more than 1,000-fold higher than healthy adult individuals [6, 16]. Indeed, FGF-23 has been proposed to be the initial adaptive response to reduced kidney function leading to low 1,25D and secondary hyperparathyroidism in CKD and to be a key initiating factor in CKD-mineral and bone disorder [17]. These high levels of circulating FGF-23 are associated with significantly worse clinical outcomes in both pre-dialysis CKD and in end-stage renal disease [16, 18, 19, 20, 21]. 

The mechanisms leading to increased FGF-23 concentrations in CKD are not clear. Increased FGF-23 concentrations do not appear to be due to decreased renal clearance only. Rather, there is evidence for increased FGF-23 production in advanced CKD [22] due to excess PTH or some unknown primary stimulus increasing FGF-23 transcription as well as factors that decrease FGF-23 catabolism. Increased serum FGF-23 levels in CKD might also occur due to end-organ resistance to the phosphaturic effect of FGF-23 mediated by an unknown feedback mechanism from kidney to bone [3]. Finally, bone turnover and mineralization per se might also regulate FGF-23 production in CKD [23]. High levels of PTH [24, 25, 26] as well as activating mutations of small G-protein G(s) α [27, 28, 29] can lead to increased FGF-23 levels. On the other hand, treatment of animal models with bisphosphonates decreases FGF-23 expression [30]. In addition, Wesseling-Perry et al. [31] found that high levels of FGF-23 were associated with improved indices of skeletal mineralization in children with high turnover renal osteodystrophy on peritoneal dialysis (PD). The same group also reported that defective skeletal mineralization was not directly related to circulating FGF-23 levels in pre-dialysis CKD children [32]. There is no report on serum levels of FGF-23 and their associations with bone histomorphometric parameters reflecting mineralization and formation in adult patients with CKD stage 5 on dialysis (CKD-5D). The present study was designed to test the hypothesis that higher FGF-23 levels are associated with improved mineralization parameters in adult CKD-5D patients. 

### Patients and methods 

This cross-sectional study evaluated the relationship between FGF-23 and bone histomorphometric parameters in CKD-5D patients. Consecutive patients on hemodialysis (HD) enrolled in the Renal Osteodystrophy Registry of the University of Kentucky were screened to participate in the study. The study was reviewed and approved by the Institutional Review Board at the University of Kentucky and was conducted according to the Declaration of Helsinki. All patients signed informed consent. 

Inclusion criteria were age ≥ 18 years, maintenance HD 3 times a week for at least 6 months, adequate HD (Kt/V ≥ 1.2), vitamin D-naïve or on steady dose of vitamin D or vitamin D analogs for ≥ 6 months, and willingness and mental competence to participate in the study. Exclusion criteria were previous renal transplantation or parathyroidectomy, pregnancy or lactation, use of calcimimetics, and medications known to affect bone metabolism such as glucocorticoids (except vitamin D), and life-threatening comorbid conditions such as HIV, malignancy, active infection, and hepatic disease. 

Anterior iliac crest bone biopsies were performed after tetracycline labeling for mineralized bone histology and histomorphometry. Blood was drawn at the time of biopsy for determinations of serum levels of calcium, phosphorus, bone specific alkaline phosphatase (BSAP), tartrate-resistant acid phosphatase-5b (TRAP5b), iPTH, 1,25D, FGF-23, and s-Klotho. 

Serum calcium and phosphorus levels were measured by automated techniques. IPTH was measured by a radioimmunometric assay (Scantibodies, Santee, CA, USA), normal range: 14 – 66 pg/ml; intra-assay coefficient of variation: < 5%. Serum BSAP levels were measured using an enzyme-linked immunosorbent assay (Metra BAP ELISA, Quidel, San Diego, CA, USA), normal range: 18 – 75 U/L; intra-assay coefficient of variation: < 6%. Serum TRAP5b levels were determined using an ELISA kit (MicroVue, Quidel, Santa Clara, CA, USA), normal range: 1.2 – 6.7 U/L; intra-assay coefficient of variation: < 2.2%. The assay for 1,25D involved extraction and subsequent purification of vitamin D metabolites from serum using C18OH cartridges. Following extraction, the treated samples were then assayed using a competitive radioimmunoassay (Heartland Assays, Ames, IA, USA) [33], normal range: 15 – 35 pg/ml; intra-assay coefficient of variation: 9.8%. Serum s-Klotho was measured using an ELISA kit (Immuno Biological Laboratories Co., Ltd., Tokyo, Japan) [34], normal range: 239 – 1,266 pg/mL; intra-assay coefficients of variation: 3.6% ± 1.5%. Serum intact FGF-23 levels were measured using a two-site ELISA kit (Kainos Laboratories, Tokyo, Japan). The two specific murine monoclonal antibodies employed bind to full-length FGF-23, without inclusion of C-terminal fragments (normal range: 18 – 108 pg/mL). The kit has a sensitivity with minimum detection limit of 3 pg/mL and a wide quantification range from 3 to 800 pg/mL. Each sample was assessed in triplicate. With concentrations > 800 pg/mL, the procedure was repeated using a 1/10 dilution. The intra- and inter-assay coefficients of variation were 4.7 and 6.4%, respectively. 

### Mineralized bone histology and bone histomorphometry 

All bone samples were processed and analyzed at the Bone Diagnostic and Research Laboratory, University of Kentucky. For double labeling of bone, patients were given oral tetracycline hydrochloride 500 mg twice daily for 2 days followed by a 10-day tetracycline-free interval and another course of tetracycline hydrochloride at the same dose for 4 days. Iliac crest bone biopsies were performed after an additional 4 days. Bone samples were 0.3 cm in diameter and at least 2.5 cm in length. They were fixed in ethanol at room temperature, dehydrated, and embedded in methyl methacrylate as described previously [35]. Sections were stained with the modified Masson-Goldner trichrome stain [36], the aurin tricarboxylic acid stain [37], and solochrome azurine stain [38]. Unstained sections were prepared for phase-contrast and fluorescence light microscopy. Bone histomorphometry for static and dynamic parameters of bone structure, formation, and resorption was done at a magnification × 200 using the Osteoplan II system [39, 40]. All measured histomorphometric parameters are in compliance with the recommendations of the nomenclature committee of the American Society for Bone and Mineral Research [41, 42]. Renal osteodystrophy was assessed by evaluation of its components “Turnover, Mineralization, and Volume” (TMV) according to Kidney Disease Improving Global Outcomes (KDIGO) recommendations [43, 44]. Bone turnover was assessed by activation frequency (Ac.f., normal range from 0.49 to 0.72/year) and bone formation rate/bone surface (BFR/BS, normal range from 1.81 to 3.80 mm^3^/cm^2^/year). Mineralization was assessed by osteoid thickness and mineralization lag time. Osteomalacia was defined as osteoid thickness > 20 µm combined with mineralization lag time > 100 days or osteoid maturation time > 40 days [35, 45]. Bone volume was assessed by cancellous bone volume/tissue volume and by trabecular and cortical thickness. 

### Statistical analyses 

Results were given as mean ± standard deviation (SD) or median and 25^th^ – 75^th^ interquartile (IQ) when values were not normally distributed. Categorical variables were expressed as percentages. Comparisons of continuous variables were evaluated using Mann-Whitney U-test. Spearman’s rank test was used to assess correlations. Receiver operating characteristic (ROC) curves were generated for determination of diagnostic values of markers of bone and mineral metabolism for normal mineralization lag time. We calculated the area under the curve (AUC) to determine the best parametric test value. Stepwise multivariate linear regression analysis was used to examine the association between bone turnover, mineralization, volume, and biochemical parameters. Independent variables for inclusion were selected using a stepwise algorithm with p ≤ 0.20 to enter the model and p ≤ 0.05 to remain in the model. The relations were presented as standardized beta-coefficients (β). Non-normally distributed parameters were log-transformed for multivariate regression analysis. All statistical analyses were performed using SPSS version 21 (SPSS, Inc, Chicago, IL, USA). A p-value ≤ 0.05 was considered statistically significant. 

## Results 

The study included a total of 36 patients; 15 men and 21 women with a mean age of 50 ± 15 years (range, 26 – 78 years). The mean duration of HD was 65 ± 36 months (range, 7 – 156 months). Causes for CKD were diabetes mellitus (22%), hypertension (30%), glomerular disease (8%), cystic kidney disease (7%), and unknown (33%). 

All subjects except 1 were treated with oral phosphate binders; 69% of patients were given calcium-containing phosphate binders and 28% of patients were given non-calcium-containing phosphate binders. 47% of patients received vitamin D analog therapy. Biochemical parameters of the study subjects are shown in Table 1. Serum concentrations of calcium were slightly decreased, serum phosphorus elevated as expected in CKD-5. All but 2 patients had very low levels of circulating 1,25D. Two patients had normal levels of circulating 1,25D; 1 was treated and 1 was untreated with vitamin D analog therapy. BSAP, TRAP5b, and iPTH were in ranges expected for patients on chronic dialysis. S-Klotho results were in the normal range while the average serum FGF-23 levels were increased 200-fold. Serum FGF-23 concentrations ranged from 18 to 37,349 pg/mL with a median value of 2,577 pg/mL. FGF-23 concentrations were in the normal range in only 3 patients. FGF-23 correlated significantly with serum phosphorus (ρ = 0.53, p < 0.01), BSAP (ρ = 0.46, p < 0.01), and iPTH (ρ = 0.39, p < 0.05) but not with TRAP5b (ρ = 0.03, ns) or s-Klotho (ρ = 0.20, ns). 

Bone turnover was low in 20 and high in 16 patients based on histomorphometric results. Patients with low bone turnover had significantly lower serum FGF-23 levels than patients with high bone turnover (median: 435 pg/mL, IQ: 195 – 2,890 vs. median: 11,073 pg/mL, IQ: 3,493 – 30,637, p < 0.001). FGF-23 correlated positively with Ac.f, BFR/BS, and osteoblast number per bone perimeter (Table 2). Among all measured biochemical parameters, phosphorus was the only independent predictor for Ac.f and BFR/BS (β = 0.76 and 0.79, respectively, p < 0.001) by univariate regression analysis (Table 2). 

**Figure 1.**
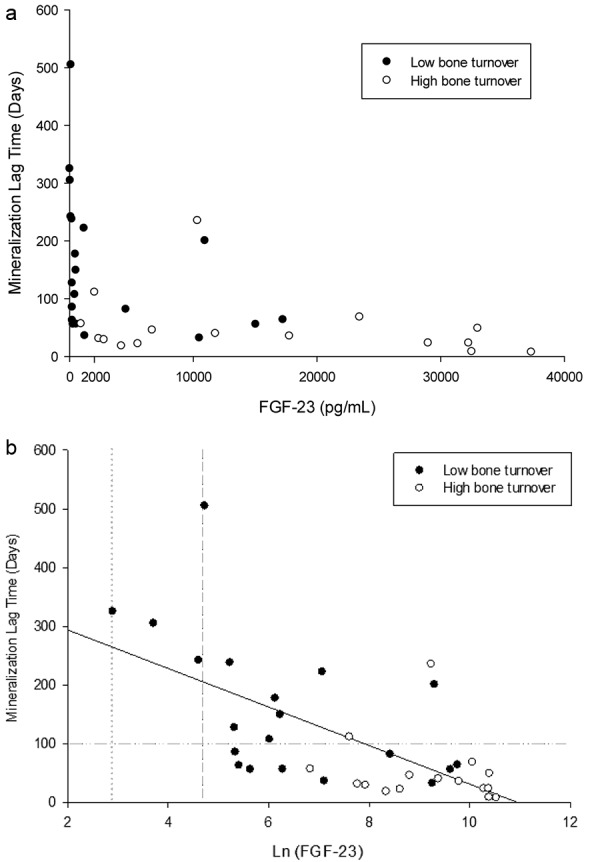
a: Scatter plot of FGF-23 (pg/mL) at different levels of mineralization lag time (days). Horizontal dotted line represents upper-normal range of mineralization lag time. b: Scatter plot of ln-transformed (Ln) FGF-23 at different levels of mineralization lag time. Black solid line represents linear regression (r^2^ = 0.41, p < 0.001). Vertical grey lines represent FGF-23 normal ranges. Grey dotted line = 18 pg/mL. Grey short dashed line = 108 pg/mL. Horizontal grey line represents mineralization lag time at 100 days.

None of the patients had osteomalacia when the appropriate histomorphometric parameters for osteomalacia were applied (see Methods section). There was a strong negative correlation between FGF-23 and mineralization lag time (ρ = –0.69, p < 0.001) and a weaker inverse correlation with osteoid maturation time (ρ = –0.46, p < 0.05) (Table 2). Normal mineralization lag time was observed in 90% of the patients with FGF-23 levels above 2,000 pg/mL (Figures 1a, b). ROC curves for prediction of normal mineralization lag time revealed that FGF-23 and phosphorus had the best predictive values (AUC: 0.85 and 0.82, respectively), superior to 1,25D, TRAP5b, iPTH, and BSAP (AUC: 0.66 – 0.78). Of note, stepwise multivariate regression analysis showed that FGF-23 was the only independent predictor for mineralization lag time (Table 3). 

Cancellous bone volume was in the osteoporotic range in 44% of patients and normal in the others. There was no difference in FGF-23 or other serum biochemical marker levels between osteoporotic and non-osteoporotic patients. However, FGF-23 correlated positively with cancellous bone volume and negatively with cortical thickness (Table 2). BSAP was the only predictor of bone volume by stepwise multivariate regression analysis (β = 0.43, p < 0.05). 

## Discussion 

Our study confirms previous findings that the circulating concentrations of FGF-23 in patients with CKD-5 span a wide range of values from normal to extremely high levels [6]. While prior studies have implicated a role for PTH and high bone turnover in stimulation of FGF-23 in bone of pediatric patients with CKD [15], our study is the first to show in adult CKD-5D an inverse correlation between mineralization lag time/osteoid maturation time and FGF-23. We found normal mineralization lag time in this population to be associated with very high levels of FGF-23 (> 2,000 pg/mL), whereas prolonged mineralization lag time was associated with less elevated FGF-23 levels. Mineralization lag time encompasses bone mineralization and bone formation, while osteoid maturation time is the only histomorphometric parameter exclusively reflecting mineralization. Using regression analysis, FGF-23 did not predict osteoid maturation time but was the only independent predictor for mineralization lag time. Alterations in bone turnover and mineralization of bone are known to regulate FGF-23 in a variety of clinical settings [3]. 

Our study showed that high bone turnover was associated with very high FGF-23 levels and low bone turnover was seen with somewhat lower FGF-23 levels. The positive correlation between FGF-23 and phosphorus, BSAP or iPTH can be explained by the relationship between FGF-23 and bone formation [3, 23]. The lack of correlation with TRAP5b indicates that FGF-23 may not be related to bone resorption. 

In contrast to our patients with CKD-5D, in subjects with normal renal function excess, FGF-23 can cause rickets/osteomalacia through renal effects to reduce serum phosphorus and 1,25D levels [7, 8, 9, 10, 11]. Our findings that impaired mineralization is associated with lower levels of FGF-23 in adult CKD-5D patients are not consistent with these reports. 

FGF-23 could directly inhibit bone formation independently of its effects on phosphorus homeostasis, as evidenced by in-vitro effects of FGF-23 overexpression to inhibit osteoblast differentiation and matrix mineralization [46]. If so, elevated FGF-23 would be expected to be associated with prolongation of the mineralization lag time, findings opposite to our current observations. Also, we did not observe any association between Klotho, bone histomorphometric measures and the other biochemical results, although s-Klotho in concert with FGF-23 has been reported to directly regulate osteoblast function in-vitro [13]. In CKD patients, Fliser et al. [47] and Komaba et al. [48] also did not find a major role of s-Klotho as an endogenous regulatory hormone or biomarker in CKD when compared to the data available on FGF-23. On the other hand, FGF-23-null mice have soft tissue calcifications, severe growth retardation, and bone mineralization abnormalities [49] that may be mediated by elevated osteopontin [12]. Of all the possible scenarios, this one is most consistent with our findings that low FGF-23 levels are associated with prolonged mineralization lag time in CKD. Further investigations are needed to understand the mechanisms linking bone formation and mineralization to FGF-23 levels in CKD patients. 

Wesseling-Perry et al. [50] studied the relationship between plasma FGF-23 concentrations and bone mineralization in pediatric CKD-5 patients on PD who displayed biochemical evidence of secondary hyperparathyroidism. Similar to our study, they demonstrated that high serum FGF-23 levels were associated with shorter osteoid maturation time and mineralization lag time. In contrast to their study, we did not find any correlation between FGF-23 levels and osteoid thickness. The main difference between our study and their study is the patient population as they studied children with secondary hyperparathyroidism who were treated with PD [50], while we studied adults on HD and included both high and low bone turnover. In a separate study by Wesseling-Perry et al. [32], no association between FGF-23 levels and abnormalities in mineralization was found in pre-dialysis CKD children. This was explained by renal excretion of FGF-23 which may affect the relationship between FGF-23 and bone histology. 

A variety of assays that are used for detecting FGF-23 may be considered as potentially complicating this issue [51]. The full length of intact FGF-23 levels can be determined with a sandwich ELISA technique, in which two kinds of monoclonal antibodies detect the simultaneous presence of both the N-terminal and C-terminal portions of FGF-23. In contrast, the C-terminal assay recognizes both full-length and processed C-terminal fragments of FGF-23. Since the vast majority of circulating FGF-23 in dialysis patients consists of the full-length intact active form of the molecule [50, 52], the intact FGF-23 assay used in our studies appears to be more biologically relevant than the C-terminal FGF-23 fragment in dialysis patients [53]. Nevertheless, values of FGF-23 by these different assays are well correlated [20, 50] and both high intact FGF-23 and C-terminal FGF-23 concentrations are consistently and independently associated with increased mortality risk and poor outcome in patients with CKD; thus, it may be considered desirable to lower circulating FGF-23 levels. Hence, reductions in PTH are associated with reduced FGF-23 levels in CKD-5 patients [54, 55]. Our study either suggests that impaired bone formation along with mineralization may lower FGF-23 or that high FGF-23 may be important for bone health. Distinguishing between these two possibilities will require determining the cause-and-effect relationship between FGF-23 and mineralization lag time. 

A potential limitation of the study is its cross-sectional design in a relatively small number of patients without the inclusion of patients with overt osteomalacia. Generalized osteomalacia is nowadays observed in only a small percentage of adult patients with CKD-5D [45]. To our knowledge, this is the first study evaluating the association between FGF-23 levels and bone histomorphometric parameters in a representative adult CKD-5D population. Further studies in adult patients are needed to evaluate FGF-23 expression in bone together with other factors potentially contributing to the regulation of bone formation and mineralization in CKD-mineral and bone disorders. 

In conclusion, in adult CKD-5D patients, FGF-23 is a unique predictor for mineralization lag time. Very high levels of FGF-23 are required for normal mineralization lag time in these patients. 

## Acknowledgments 

Research reported in this publication was supported by National Institute of Arthritis and Musculoskeletal and Skin Diseases, National Institutes of Health Award R01080770 and the Kentucky Nephrology Research Trust. The content is solely the responsibility of the authors and does not necessarily represent the official views of the National Institutes of Health. 

## Conflict of interest 

None. 

**Table 1.** Serum biochemical parameters of the study population.

Total n = 36	Mean ± SD or median (25 – 75 IQ)	Reference values
Calcium (mg/dL)	9.1 ± 0.8	8.5 – 10.3
Phosphorus (mg/dL)	4.9 (4.2 – 7.0)	2.5 – 4.5
BSAP (U/L)	56 (31 – 137)	18 – 75
TRAP5b (U/L)	6.4 (4.0 – 10.7)	1.2 – 6.7
iPTH (pg/mL)	425 (67 – 923)	14 – 66
1,25D (pg/mL)	4.2 (3.6 – 5.3)	15 – 35
FGF-23 (pg/mL)	2,577 (347 – 13,422)	18 – 108
Soluble Klotho (pg/mL)	538 ± 208	239 – 1,266

SD = standard deviation; IQ = interquartile range; BSAP = bone specific alkaline phosphatase; TRAP5b = tartrate-resistant acid phosphatase-5b; iPTH = intact parathyroid hormone; 1,25D = 1,25 dihydroxyvitamin D; FGF-23 = fibroblast growth factor-23.

**Table 2.** Correlation coefficients (ρ) between serum biochemical markers and bone histomorphometric parameters (Spearman’s test).

Total n = 36	FGF-23	iPTH	BSAP	TRAP5b	Phosphorus
Turnover					
Activation frequency (y^–1^)	0.60**	0.66**	0.71**	0.58**	0.64**
Bone formation rate/bone surface (mm^3^/cm^2^/y)	0.57**	0.71**	0.75**	0.64**	0.66**
Osteoblast number/bone perimeter (N/mm)	0.38*	0.73**	0.71**	0.58**	0.62**
Osteoclast number/bone perimeter (N/mm)	0.28	0.69**	0.62**	0.48*	0.46**
Mineralization					
Lamellar osteoid thickness (µm)	0.08	0.67**	0.75**	0.65**	0.42*
Osteoid maturation time (Days)	–0.46**	0.03	–0.05	0.18	–0.21
Mineralization lag time (Days)	–0.69**	–0.31	–0.36*	–0.12	–0.51**
Volume					
Bone volume/tissue volume (%)	0.38*	0.25	0.42*	0.21	–0.04
Trabecular thickness (µm)	0.32	0.09	0.28	0.08	–0.06
Cortical thickness (µm)	–0.60**	–0.43*	–0.31	–0.15	–0.46*

*p < 0.05 (2-tailed); **p < 0.01. (2-tailed).

**Table 3.** Multivariate and univariate regression analysis of mineralization lag time.

Dependent variables:	Mineralization lag time			
	Unstandardized coefficients		Standardized coefficients	
	β-value	SE	β-value	p-value
Predictors multivariate regression analysis:				
FGF-23*	–34.71	9.89	–0.70	0.00
iPTH*	17.92	19.64	–0.57	0.37
BSAP*	46.10	32.45	0.45	0.17
TRAP5b*	–81.10	40.47	–0.47	0.06
Phosphorus*	–177.81	91.02	0.29	0.06
Predictor univariate regression analysis:				
FGF-23*	–32.89	8.39	–0.60	0.00

*Log-transformed performed before analysis. SE = standard error.
